# B and T Cell Responses to SARS-CoV-2 Vaccination in Kidney and Liver Transplant Recipients with and without Previous COVID-19

**DOI:** 10.3390/v16010001

**Published:** 2023-12-19

**Authors:** Christina Watschinger, Gerald Stampfel, Andreas Zollner, Anna M. Hoog, Annika Rössler, Silvia Reiter, Kristina Dax, Janine Kimpel, Herbert Tilg, Marlies Antlanger, Elisabeth Schwaiger, Alexander R. Moschen

**Affiliations:** 1 Department of Internal Medicine 2 (Gastroenterology and Hepatology, Endocrinology and Metabolism, Nephrology, Rheumatology), Faculty of Medicine, Johannes Kepler University Linz, 4021 Linz, Austria; 2 Christian Doppler Laboratory for Mucosal Immunology, Faculty of Medicine, Johannes Kepler University Linz, 4021 Linz, Austria; 3 Department of Medicine, Division of Internal Medicine 1 (Gastroenterology and Hepatology, Endocrinology and Metabolism), Medical University of Innsbruck, 6020 Innsbruck, Austria; 4 Department of Hygiene, Microbiology, and Public Health, Institute of Virology, Medical University of Innsbruck, 6020 Innsbruck, Austria; 5 Department of Internal Medicine, Brothers of Saint John of God Eisenstadt, 7000 Eisenstadt, Austria

**Keywords:** SARS-CoV-2, COVID-19, vaccination, liver transplantation, kidney transplantation, antibody response, T cell response

## Abstract

(1) Background: Vulnerable populations including transplant recipients are jeopardised by COVID-19. Herein, we report on B and T cell responses among liver and kidney organ recipients at our centre. (2) Methods: 23 liver and 45 kidney (14 thereof combined kidney/pancreas) transplanted patients were vaccinated with two doses of BNT162b2 followed by a booster dose of mRNA-1273 in 28 non-responders 4 months thereafter. Anti-SARS-CoV-2-Ig was measured by specific ELISA and virus neutralisation assay; T cell responses were measured by a spike protein-specific IFN-γ release assay. (3) Results: Compared to controls, B and T cell responses were weak in transplant recipients, particularly in those without prior exposure to SARS-CoV-2. Within this group, only 15% after the first and 58.3% after the second vaccination achieved seroconversion. A total of 14 out of 28 vaccination non-responders achieved a seroconversion after a third dose. Vaccination side effects were more frequent in healthy controls. The use of mycophenolate was associated with reduced anti-SARS-CoV-2-Ig production. (4) Conclusions: Our data confirm that vaccination responses are insufficient after standard vaccination in liver and kidney transplant recipients and are affected to a variable degree by specific immunosuppressants, particularly mycophenolate. Monitoring vaccination success and re-vaccinating those who are unresponsive seems prudent to achieve sufficient titres. Overall, prospective large-scale, multinational, multicentre studies or high-quality meta-analyses will be needed to generate personalised vaccination strategies in order to achieve protective immunity in high-risk, hard-to-immunize populations.

## 1. Introduction

The emergence of solid organ transplantation (SOT) in the mid-fifties introduced a lifesaving and durable treatment option for a myriad of patients suffering from end-stage organ failure such as of the kidney or the liver [1]. Besides significant surgical advances, it was the induction of immunosuppression that prevented acute and chronic rejection and prolonged allograft survival [2].

Despite excellent long-term median transplant survival rates—more than 14 years for both kidney and liver [3]—solid organ transplantation comes at a price mostly driven by the need for long-term immunosuppression. Complications thereof include predisposition to infections, malignancies, and cardiovascular diseases [4].

Infectious complications are an important cause of morbidity and mortality in solid organ transplant recipients [5]. The COVID-19 pandemic, with different variants of the acute respiratory syndrome coronavirus 2 (SARS-CoV-2) as the causative agent, has challenged the field of SOT in various aspects [6].

SOT recipients were thought to be at increased risk for severe COVID-19 due to immunosuppression and comorbidities, although this apprehension has been relativised by recent reports. A study from Portugal compared mortality rates in >2000 kidney and kidney–pancreas transplant recipients between two periods, the first between September 2012 and March 2020 and the second between March 2020 and August 2022. The authors identified a small, yet significant, increase in both absolute and relative excess mortality associated with COVID-19, which is mainly attributable to the second seasonal peak of the disease [7]. In contrast, an analysis from the Spanish National Registry of Hospital Discharges including >117,000 adults hospitalised for COVID-19 in 2020 did not identify increased mortality among the subgroup of 491 SOT recipients after adjusting for baseline characteristics, suggesting that co-morbidities are the risk drivers for both the general and the SOT population [8]. Concordantly, a recent large meta-analysis including >1500 COVID-19-affected liver transplant (LT) recipients identified similar outcomes compared to non-LT patients with COVID-19 [9].

Numerous studies have shown that SOT recipients are less likely to mount protective immunity to COVID-19-specific vaccines. A recent meta-analysis compiled the results from 91 reports involving >11,800 patients after SOT. The humoral immune response was as low as 9.5% after the first dose and 43.6% after the second dose. A booster vaccination increased the response rates to 55.1%, yet a considerable number of recipients did not build up any protective immunity [10]. Comparable data come from another large systematic review and meta-analysis including >11,000 recipients of solid organ transplants, with anti-spike antibody positivity of 10.4% for one, 44.9% for two, and 63.1% for three doses of mRNA vaccine. The authors concluded that protective immunity remains low despite multiple dosing [11]. Nevertheless, the overall COVID-19 vaccination of transplant recipients seems effective as hospitalisation rates markedly decreased with an increasing number of doses in later waves [12,13].

Herein, we contribute additional evidence of vaccination outcomes in terms of B and T cell immunity from a single centre cohort of 68 liver and kidney transplant recipients receiving two doses of BNT162b2 (Pfizer-BioNTech, Mainz, Germany) and a booster with mRNA-1273 (Moderna Inc., Cambridge, MA, USA).

## 2. Materials and Methods

### 2.1. Study Participants

In this study, we compared humoral and cellular immune responses after the application of the SARS-CoV-2 mRNA vaccine from Pfizer-BioNTech (BNT162b2) in kidney (*n* = 31), combined kidney and pancreas (*n* = 14), and liver transplant recipients (LTX, *n* = 23). Kidney and kidney and pancreas recipients were combined into one subgroup and designated as NTX + PTX throughout this study. A total of 8 out of 68 SOT patients (all within the NTX + PTX group) had a history of SARS-CoV-2 infection (confirmed by past PCR or baseline ELISA positivity). All subjects received two vaccinations with BNT162b2 at an interval of 4 to 6 weeks between March and April 2021. In total, 28 patients who failed to respond to two injections of the Pfizer-BioNTech vaccine BNT162b2 received a booster vaccination 15 weeks after the second vaccination using the mRNA-1273 from Moderna. A total of 41 in-house healthcare professionals served as a healthy control comparator group. Details are given in an earlier report [14]. This study was approved by the ethics committee of the Johannes Kepler University Linz (EC-No. 1322/2020) and written informed consent was obtained from all study participants.

### 2.2. Assessment of Vaccine-Related Side Effects

A detailed evaluation of vaccine-related side effects was carried out for all treated persons after the second vaccine dose.

### 2.3. Procedures

Antibodies against the spike protein were analysed using a SARS-CoV-2-QuantiVac ELISA (Euroimmun, Lübeck, Germany). ELISA antibody concentrations are reported in relative units (RU)/mL. RU/mL are convertible to international units (BAU/mL) by multiplying it with the factor 3.2. Values below the threshold of 8 RU/mL were considered negative.

For neutralisation assays, the vesicular stomatitis (VSV) micro neutralisation test (VSV-MNT) was deployed as previously described [14,15].

The presence of SARS-CoV-2 specific T cells directed against the spike (S) protein was assessed four weeks after two immunisation doses (day 49) measuring IFN-γ release from spike glycoprotein (pepS) stimulated T cells as previously described [14].

### 2.4. Statistical Analysis

Data are presented as median [25th–75th percentile] if not otherwise specified.

ELISA data were log-transformed for visualisation. Zero values were set to 0.1 to allow log transformation. Differences between groups were analysed using Kruskal–Wallis ANOVA followed by Dunn’s multiple comparison. For paired linear data, a Friedman test followed by Dunn’s multiple comparison was used. Categorical variables were analysed using the chi-square (χ^2^) test. *p*-values below 0.05 were considered statistically significant and below 0.01 were highly significant. Statistical tests were performed using SPSS version 22 (SPSS, 2016). All statistical analysis was supervised by a professional statistician.

## 3. Results

### 3.1. Baseline Demographics

A total of 68 SOT recipients were included in this study, comprising 45 (66.2%) kidney transplant recipients (including 14 with combined kidney and pancreas transplants) and 23 (33.8%) liver transplant recipients (Table 1). IgG titres SARS-CoV-2 at day 0, 21, and 49 as well as side effects related to the second vaccination were compared to 41 healthy control subjects. Detailed results and discussion of such is subject to an earlier report [14]. Baseline characteristics are summarised in Table 1.

### 3.2. Vaccination with BNT162b2 Results in Reduced Humoral and Cellular Immune Responses in SOT Recipients

**Figure 1:**
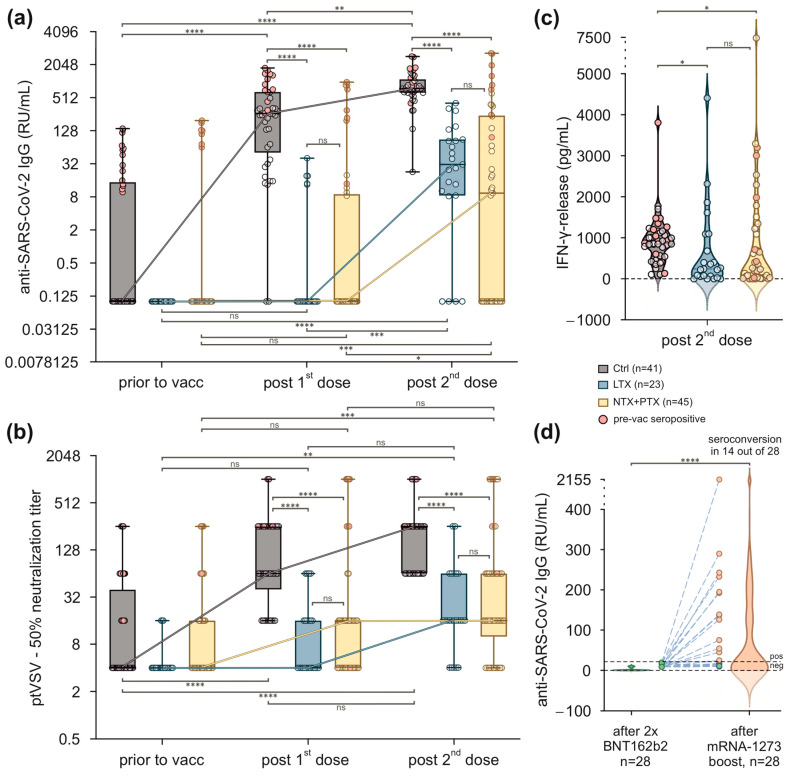
Vaccination with BNT162b2 results in reduced humoral and cellular responses in SOT recipients. (**a**) Anti-SARS-CoV-2 IgG titres (RU/mL) in healthy controls, LTX, and NTX + PTX recipients. Each triplet represents data within the indicated timepoints before and after vaccination. Participants with a positive history of COVID-19 prior to vaccination (pre-vac seropositive) are indicated in red. Values below the assay’s threshold of 8 RU/mL were set at 0.1 RU/mL. Data were log2 transformed. (**b**) Functional testing of antibodies using spike-pseudotyped vesicular stomatitis viruses (ptVSV). Titres ≤ 1:4 were considered negative. Each triplet represents data from the indicated timepoints before and after vaccination. (**c**) SARS-CoV-2 spike protein-specific T cells were studied by interferon-γ release assay (IGRA) in the indicated groups after two vaccinations at day 49. (**d**) Anti-SARS-CoV-2-Ig after booster vaccination with mRNA-1273. Combined violin- and scatterplots of SARS-CoV-2 IgG titres in SOT recipients after the second vaccination and after the third booster vaccination. A total of 14 out of 28 SOT recipients achieved a seroconversion. (**a**,**b**) Box plots represent values as median (bold horizontal line), 75% confidence interval (box), and minimum and maximum values (whiskers). Dot clouds represent individual values, and the numbers are indicated. * *p* < 0.05; ** *p* < 0.01; *** *p* < 0.001; **** *p* < 0.0001; paired data were analysed by Friedman test followed by Dunn’s multiple comparison. (**c**,**d**) Each violin plot represents the distribution of IFN-γ (pg/mL) for individual groups, with the dashed line indicating the median and the dotted lines representing 75% confidence intervals. Each data point represents one participant. Kruskal–Wallis ANOVA followed by Dunn’s multiple comparison test. (**a**–**d**) Crtl = healthy control; LTX = liver transplant recipients; NTX + PTX = kidney + kidney/pancreas recipients.

Several studies indicate that SOT recipients exhibit an impaired immune response to SARS-CoV-2 vaccines. Regarding antibody responses in liver transplant patients, we did not observe a significant increase in anti-SARS-CoV-2 IgG levels after the first vaccination (0.1 [0.1–0.1] RU/mL) and a small but significant increase 4 weeks was observed after the second vaccination (31.2 [8.5–88.9] RU/mL, *p* < 0.0001). This corresponded to a seroconversion rate of 21.7% (5 out of 23) after the first and 82.6% (19 out of 23) after the second vaccination. Accordingly, in patients after kidney or combined kidney and pancreas transplantation, antibody concentrations were 0.1 [0.1–9] RU/mL after the first and increased significantly to 9.4 [0.1–241.9] after the second vaccination (*p* < 0.001). This corresponded to a seroconversion rate of 26.6% (12 out 45) after the first and 53.3% (24 out of 45) after the second vaccination. Starting from comparable baseline concentrations, both numerically and qualitatively, the antibody responses were significantly lower than observed in our control group that achieved median antibody concentrations of 263 [51.2–649.8] RU/mL after the first and 756.9 [630.4–1103.0] RU/mL after the second vaccination. The seroconversion rates were 95.1% (39 out of 41) after one and 100% (41 out of 41) after two injections of BNT162b2. Notably, vaccination responses were improved in kidney/pancreas transplant recipients and healthy controls who had a positive history of COVID-19 (Figure 1a).

Antibody concentrations as determined by RDB ELISA were complemented by a neutralisation assay using an ancestral variant spike-pseudotyped vesicular stomatitis virus (VSV). At large, results retrieved from the neutralisation assays mirrored the data obtained from the RDB ELISA. Median 50% neutralisation titres increased from baseline 4.0 [4.0–4.0] to 4.0 [4.0–16.0] in LTX and from baseline 4.0 [4.0–16.0] to 16.0 [4.0–16.0] in NTX + PTX patients after the first vaccination. This increase was statistically not significant in both SOT groups. Compared to baseline, following the second injection 50% neutralisation titres increased significantly to 16.0 [16.0–64.0] in LTX (*p* < 0.01) and 16.0 [10.0–64.0] in NTX + PTX subjects (*p* < 0.001). This corresponded to a seroconversion rate (cut-off titre was assumed > 1:4) of 78.3% (18 out of 23) in the LTX and 75.6% (34 out of 45) in the NTX + PTX group. Again, this increase was significantly lower than in healthy controls (Figure 1b).

To evaluate cellular immunity, interferon-γ-release assays (IGRA) were performed in peripheral blood cells 4 weeks after the second immunisation. Whereas a median IFN-γ concentration of 872.1 [551.3–1164] pg/mL was reached in healthy subjects, IFN-γ release was significantly lower in patients after liver and after kidney +/− pancreas transplantation, with median IFN-γ concentrations of 241.7 [58.8–1089] pg/mL (*p* < 0.05) and 263.2 [34.2–1241] pg/mL (*p* < 0.05), respectively (Figure 1c).

### 3.3. Transplant Patients Who Failed to Achieve Protective Antibody Titres after Two Immunisations with BNT162b2 Benefit from a Booster Vaccination with mRNA-1273

To overcome a potentially detrimental lack of protection after two injections with BNT162b2, non-responders were offered a booster vaccination with the mRNA-1273 15 weeks after the second injection. In total, 28 patients received a third dose, and serum anti-SARS-CoV-2 Ig concentrations were assayed 6 weeks thereafter. As outlined in Figure 1d, median antibody concentrations increased from 0.1 [0.1–4.7] at the timepoint of the third injection to 11.8 [1.7–129.7] 6 weeks thereafter (*p* < 0.0001). It is noteworthy to mention that seroconversion was achieved in 50% (14 out 28) of SOT patients after a third booster injection.

### 3.4. In SOT Recipients, Humoral Responses after Two Vaccinations with BNT162b2 Are Most Negatively Affected by Mycophenolate

Several studies indicate that vaccination responses are reduced in patients after SOT, including immunisation against SARS-CoV-2 [16]. Among others, one reason for this reduced immune reactivity can be explained by the exposure to immunosuppressive drugs that are required to prevent rejection after transplantation. The consequent analyses were performed in a combined SOT group comprising LTX and NTX+PTX recipients. As outlined in Table 1, the majority of our SOT patients, namely 84%, received calcineurin inhibitors (CNI), alone or in combination with other immunosuppressants.

**Figure 2:**
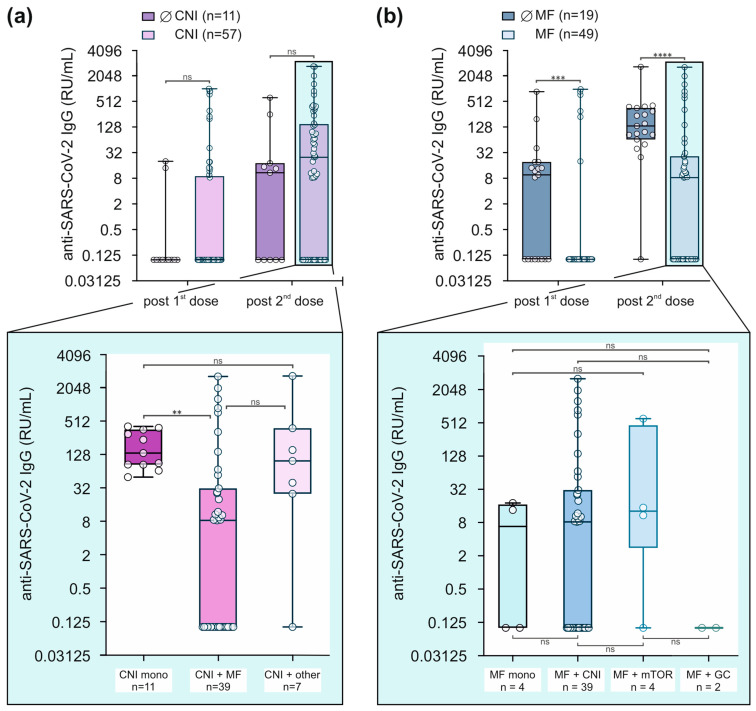
Mycophenolate use drives humoral non-response in SOT patients after SARS-CoV-2 vaccination. (**a**,**b**) Anti-SARS-CoV-2 IgG titres (RU/mL) in SOT recipients with or without CNI (A) or with or without MF (B) treatment. Each pair of bars represents data from the indicated timepoints. Highlighted and zoomed-out data illustrate anti-SARS-CoV-2-Ig concentrations in additional subsets of SOT recipients on day 49. Data were log2 transformed. Box plots represent values as median (bold horizontal line), 75% confidence interval (box), and minimum and maximum values (whiskers). Dot clouds represent individual values, and the total numbers are indicated. Values below the assay’s threshold of 8 RU/mL were set at 0.1 RU/mL. ** *p* < 0.01; *** *p* < 0.001; **** *p* < 0.0001; groups were compared using Mann–Whitney-U or Kruskal–Wallis-ANOVA followed by Dunn’s multiple comparison.

To evaluate the impact of CNI on post-vaccination anti-SARS-CoV-2 IgG concentrations, we compared 11 patients without CNI and 57 patients with CNI (Figure 2a). In patient groups without and with CNI, median anti-SARS-CoV-2 IgG concentrations were not different after the first (0.1 [0.1–0.1] versus 0.1 [0.1–9] RU/mL) and after the second immunisation (10.9 [0.1–18.2] versus 24.9 [0.1–147.7] RU/mL). When CNI-exposed patients were further divided into 11 patients on CNI monotherapy, 39 patients on CNI in combination with mycophenolate (MF), and 7 patients on CNI with other combinations (Figure 2a, lower panel), anti-SARS-CoV-2 IgG concentrations were significantly lower in the CNI + MF group compared to CNI mono patients (8.3 [0.1–31.2] versus 134.7 [83.3–357.3] RU/mL, *p* = 0.0019).

Thus, we next separated our SOT cohort into 19 patients with and 49 patients without MF in their pre-medication. As demonstrated in Figure 2b, serum anti-SARS-CoV-2 Ig concentrations were higher in patients without MF compared to patients receiving MF, both after the first (9.6 [0.1–19.3] versus 0.1 [0.1–0.1] RU/mL, *p* = 0.0006) and after the second vaccination (134.7 [65.1–357.3] versus 8.3 [0.1–26.2] RU/mL, *p* < 0.0001). Intriguingly, after subdividing MF patients into 4 groups—MF as a monotherapy, 39 patients who received MF in combination with CNI, 4 patients in combination with an mTOR inhibitor, and 2 patients in combination with corticosteroid (Figure 2b, lower panel)—no significant differences were seen between these groups. The results on circulating antibody concentrations were not mirrored by IFN-γ release.

### 3.5. SOT Recipients Experience Fewer but More Severe Vaccine-Related Side Effects Than Healthy Controls

Vaccine-related side effects were reported more frequently by healthy subjects than by SOT recipients (75.6% vs. 48.5%, *p* < 0.01). Also, systemic side effects such as fever, joint pain, nausea, and headache were more prevalent among healthy controls than SOT recipients (65.9% vs. 32.4%, *p* < 0.01, Table 1). Local reactions at the injection site such as swelling and tenderness occurred at similar frequencies (32.4% vs. 36.6%, *p* = 0.7). While fewer vaccine-related side effects were reported by SOT recipients, those effects were on average more severe (mean 0.50 vs. 0.07 on a scale between 0 and 2, *p* < 0.01). We did not observe any significant differences in vaccine-related side effects between kidney and liver transplant patients. Data are outlined in Table 1.

## 4. Discussion

Herein, we report on the vaccination results within a single-centre cohort of liver and kidney transplant recipients who are followed up at the Department of Internal Medicine 2 at the Kepler University Hospital, Linz, Austria. All SOT patients initially received the BNT162b2 vaccine in early 2021, and non-responders were subsequently boosted with mRNA-1273. Vaccination success was compared to a local healthy control cohort recruited from in-house healthcare professionals who were the subject of an earlier report [14]. Generally, both humoral and cellular immune responses were weak in SOT patients. The use of mycophenolate, as a monotherapy or in combination, was a major driver of vaccination failure. Exactly 50% of vaccinated non-responders benefited from a third booster vaccination.

SOT recipients are known to be prone to vaccination-preventable diseases, such as influenza and varicella zoster virus infections, which are associated with morbidity and mortality [17]. Thus, it is recommended that the vaccination status should be reviewed and updated during the pre-transplant setting [18]. However, antigen drifts as in the case of influenza require regular immunisations after transplantation, and the COVID-19 pandemic has presented unparalleled challenges, particularly in this aspect.

Vaccines against SARS-CoV-2 such as BNT162b2 or mRNA-1273 have proven to mount excellent immunogenicity in the general immunocompetent population [19,20]. However, SOT recipients have been shown to be less likely to develop specific immunity against SARS-CoV-2 vaccines [10,11]. Sakuraba et al. summarised 44 observational studies including 6158 SOT recipients who received two doses of BNT162b2 or mRNA-1273 and found that serologic response rates were as low as 8.6% and 34.2%, respectively [21]. In our cohort of SOT recipients, a protective humoral immune response was achieved in 82.6% of liver and 53.3% of kidney transplant recipients, resulting in an overall 52.9% seroconversion upon two immunisations with the BNT162b2 (Pfizer-BioNTech) vaccine. In contrast, healthy controls reached protective immunity in 92% after the first dose and 100% after two vaccinations. Of note and in line with previous reports [14,22], all SOT patients previously exposed to SARS-CoV-2 were efficiently immunised despite their organ transplantation and concomitant immunosuppression. Despite a good correlation between anti-spike Ig and neutralising antibodies [23], numerous studies have shown that neutralising antibody titres correlate with immune protection from COVID-19 [24]. In our cohort, neutralising antibody titres were determined and the results correlated well with those retrieved from the spike protein-specific ELISA.

Besides antibody production, T cell responses are likely to contribute to the protection against morbidity and mortality of COVID-19 [25]. Several studies demonstrated that cellular immune responses are markedly reduced after vaccination [26,27]. In immunocompromised SOT recipients, specific T cell activity was limited, and after vaccination with BNT162b2, only 56.3% were reported to elicit a sufficient response as measured using IFN-γ ELISpot assays [27]. Our study is confirmative as SOT recipients also demonstrated significantly reduced cellular immune responses.

Given the high percentage of patients not responding to two doses of BNT162b2 in our cohort, we planned to offer a short-term third injection to those who remained seronegative [28]. As at that time some data—including our own data from healthcare professionals—suggested that the mRNA-1273 vaccine might produce more robust immunity [29], we decided to offer a boost using a heterologous regime with mRNA-1273. Hall et al. presented a placebo-controlled trial using mRNA-1273 in 120 SOT recipients. Only 55% of those receiving the mRNA-1273 vaccine achieved anti-RBD antibody levels of at least 100 U per millilitre. The third dose increased the median % virus neutralisation to 71% in the mRNA-1273 group [30]. A large meta-analysis presented by Bailey AJ et al. also suggested that almost half of transplant recipients who failed to respond to two vaccinations can benefit from a third one [31]. These findings are mirrored by the data from our cohort, where half of the non-responders to basic vaccination achieved seroconversion after a third injection.

In search for factors associated with poor seroconversion rates in patients after SOT, Li J. et al. recently presented the results from a meta-analysis and identified immunosuppressive medication as a major determinant [31]. In our cohort of patients after SOT, exposure to MMF most strongly suppressed anti-SARS-CoV-2 Ig formation. Meanwhile, several studies addressed this topic and are in line with our data that MMF seems to be a relevant factor driving vaccination failure [32,33,34].

## 5. Conclusions

In conclusion, and in line with previously published data on the efficacy of currently available SARS-CoV-2 specific mRNA vaccines, our results suggest that serological testing is particularly essential in identifying non-converted SOT patients after COVID-19 vaccination. A combination of complementary testing strategies including RDB-ELISA, neutralisation assays on the B cell, and interferon-γ release assays on the T cell axis provides a holistic picture of individual immune responses. By combining such findings with high-quality clinical data, we demonstrate the specific contributions of various immunosuppressants and identify mycophenolate as a major driver of non-responsiveness. The generation of generally accepted, personalised vaccination recommendations—also in the prospect of forthcoming pandemics—will require both prospective large-scale, multinational, multicentre studies in the long run, and meta-analyses based on high-quality studies in the short run.

## Figures and Tables

**Table 1:** Baseline characteristics of study population and vaccine-related side effects.

Baseline Characteristics					
	Unit	All SOT Recipients	NTX + PTX Recipients	LTX Recipients	Healthy Controls
*N*		68	45	23	41
Post COVID, *N* (%)		8 (11.8)	8 (17.8)	0 (0)	14 (34.1)
Sex female, *N* (%)		21 (30.9)	15 (33.3)	6 (26.1)	21 (51.2)
Age, *N* (SD)	years	58.5 (18.6)	58.4 (11.2)	58.8 (28.2)	36.7 (10.3)
BMI, mean (SD)	kg/m^2^	26.0 (4.0)	26.0 (4.0)	26.2 (4.0)	23.6 (5.9)
Time since Tx, mean (SD)		13.7 (6.5)	13.9 (6.1)	13.3 (7.2)	-
Hb, mean (SD)	g/dL	13.2 (2.2)	13.0 (2.4)	13.7 (1.6)	-
GFR, mean (SD)	mL/min	52.1 (22.4)	51.7 (24.9)	52.9 (15.2)	-
GOT, mean (SD)	U/I	23.8 (7.2)	22.6 (6.0)	26.3 (9.1)	-
GPT, mean (SD)	U/I	22.2 (10.0)	21.1 (9.4)	24.8 (11.2)	-
Bilirubin, mean (SD)	mg/dL	0.6 (0.3)	0.6 (0.3)	0.6 (0.2)	-
Albumin, mean (SD)	mg/dL	4215.9 (627.4)	4233.1 (322.1)	4175.3 (1059.1)	-
Immunosuppressants, *N* (%)					
GC		19 (28)	16 (36)	3 (13)	-
CNI		57 (84)	37 (82)	20 (87)	-
MMF/MPA		49 (72)	37 (82)	12 (52)	-
AZA		3 (4)	3 (7)	0 (0)	-
mTOR		6 (9)	5 (11)	1 (4)	-
Other		1 (1)	1 (2)	0 (0)	-
**Vaccine related side effects**					
	*p*-Value	All SOT recipients	NTX + PTX recipients	LTX recipients	Healthy controls
*N*	<0.01	68	45	23	41
Any, *N* (%)	<0.01	33 (48.5)	20 (44.4)	13 (56.5)	31 (75.6)
Local, *N* (%)	0.65	22 (32.4)	14 (31.1)	8 (34.8)	15 (36.6)
Systemic, *N* (%)	<0.01	22 (32.4)	14 (31.1)	8 (34.8)	27 (65.9)
Severity, mean	<0.01	0.50	0.47	0.57	0.07

SOT = solid organ transplant; NTX = kidney transplant recipient; PTX = pancreas transplant recipient; LTX = liver transplant recipient; BMI = body mass index; Tx = transplantation; Hb = hemoglobin; GFR = estimated glomerular filtration rate (CKD-EPI); GOT = glutamic oxaloacetic transaminase; GPT = glutamic-pyruvic transaminase; CNI = calcineurin inhibitor; MMF = mycophenolate mofetil; MPA = mycophenolic acid; mTOR = mammalian target of rapamycin. Severity of experienced side effects was assessed on a scale between 0 (weak) and 2 (strong). *p*-values indicate differences between all SOT recipients and healthy controls and are calculated using the chi-square (χ^2^) test. The local side effects assessed were pain and swelling at the vaccine injection site, and systemic side effects assessed were headache, fever and chills, nausea and vomiting, joint pain, and fatigue.

## Data Availability

Deidentified data are available to other researchers for use in independent scientific research after a justified request (alexander.moschen@jku.at).
